# Geometric Morphometric Analysis of Hard and Soft Tissue in Class III Malocclusion Before and Near-End Orthodontic Treatment

**DOI:** 10.3390/jcm15020639

**Published:** 2026-01-13

**Authors:** Albert Koay Quan Hong, Neo Joe, Helmi Mohd Hadi Pritam, Khairil Aznan Mohamed Khan, Rama Krsna Rajandram, Murshida Marizan Nor

**Affiliations:** 1 Department of Family Oral Health, Faculty of Dentistry, Universiti Kebangsaan Malaysia, Jalan Raja Muda Abdul Aziz, Kuala Lumpur 50300, Malaysia; 2 School of Health Sciences, USM Health Campus, Kelantan 16150, Malaysia; 3 Department of Oral and Maxillofacial Surgery, Faculty of Dentistry, Universiti Kebangsaan Malaysia, Jalan Raja Muda Abdul Aziz, Kuala Lumpur 50300, Malaysia

**Keywords:** Class III malocclusion, geometric morphometric analysis, orthodontic treatment, skeletal pattern, hard tissue, soft tissue

## Abstract

**Background/Objectives**: Geometric morphometric analysis (GMA) is a statistical method that captures and quantifies shape variation. This study aimed to assess hard and soft tissue shape variations and changes following orthodontic treatment in Class III skeletal malocclusion using GMA. **Methods**: A retrospective study was conducted on 84 lateral cephalometric radiographs (pre-treatment and near-end treatment) of Class III patients aged 16–40 years (ANB < 2°). Thirty-five landmarks were digitized in Cartesian coordinates using MorphoJ software for shape analysis. **Results**: The sample included 62% females and 38% males, with a mean age of 24.7 ± 5.2 years. Vertical dimension variations (hypodivergent to hyperdivergent) contributed most to shape changes PC1 (23.35%), followed by anteroposterior variations PC2 (13.51%). Gender significantly influenced hard and soft tissue variation with 30.91%SS (F = 56.99, *p* < 0.0001). Males had significantly larger and longer ramus, body of the mandible, alveolar height, LAFH, TAFH and upper lip length. (PD: 0.026, *p* < 0.05). Significant shape changes were seen in the mandible (PD = 0.018, *p* < 0.05). SNB increased by 0.41° (from 81.73° ± 3.67°), and ANB improved by 0.46° but remained Class III (−0.33° ± 1.82°). Lower anterior facial height increased by 1.78 mm (*p* < 0.05). The lower incisors retroclined significantly (from 92° ± 8.56° to 87° ± 6.96°, *p* < 0.05), while the interincisal angle increased by 5.9°. Upper incisors remained procline (118° ± 11°, *p* > 0.05). Upper lip length increased by 0.4 mm (*p* < 0.05). **Conclusions**: Vertical and anteroposterior shape variations are notable within Class III malocclusion. Post-treatment changes in both hard and soft tissues indicate that orthodontic camouflage can enhance facial esthetics and skeletal balance. GMA provides objective quantification and visualization of these treatment-related craniofacial changes.

## 1. Introduction

Class III malocclusion is defined as the lower incisor edge lying anterior to the cingulum plateau of the upper incisors. The prevalence of Class III malocclusion varies significantly among and within populations, from 1% to more than 10%. The greatest incidence is found among Asian people [1], especially in the Chinese (15.69%) and Malaysian (16.59%) populations [2]. Nevertheless, the Indian population shows a relatively lower prevalence as compared to other races [2]. The etiology of Class III malocclusion is multifactorial, involving complex interactions between genetic and environmental factors. Skeletal presentations may include mandibular prognathism, maxillary retrognathism, or a combination of both [1], and functional or “pseudo” Class III malocclusion may occur due to centric relation–centric occlusion discrepancies [3]. The facial profile is concave and this significantly impacts facial esthetics [4]. The upper incisors are usually proclined and the lower incisors can either be retroclined, upright or proclined, depending on the amount of dentoalveolar compensation. The lower lips are usually full or pendulous [5], while the upper lips may be short, retruded or flat resulting in an acute nasolabial angle [6]. Variations in hard and soft tissue craniofacial characteristics within Class III malocclusion were observed due to factors such as patients’ growth patterns [7], gender [8], age [9] and race [10].

The diagnosis and management of Class III malocclusion remain clinically challenging due to this morphological heterogeneity and the frequent coexistence of vertical skeletal discrepancies, which further complicate treatment planning [11]. The available treatment options include growth modification through orthopedic appliances, camouflage treatment using orthodontic dentoalveolar compensation and orthognathic surgery. For non-growing adults with mild to moderate skeletal Class III discrepancies and satisfactory facial esthetics, orthodontic camouflage is an effective option [12], whereas severe skeletal discrepancies generally require surgical correction [13]. Therefore, a thorough understanding of the anteroposterior and vertical skeletal relationships, along with dentoalveolar adaptations, is crucial when planning treatment for Class III malocclusion.

Orthodontic intervention can produce notable alterations in soft tissue that affect facial esthetics and function. In orthodontic camouflage, the lower incisors are retroclined, while the upper incisors are proclined to achieve a positive overjet, which results in improvements in lower lip protrusion and upper lip length [4,14]. Failure to adequately anticipate these changes may lead to an inaccurate prognosis or misjudgment of case complexity. Consequently, objective methods that allow visualization and quantification of craniofacial changes may enhance individualized treatment planning and clinician–patient communication [15].

Cephalometric radiography is the most common skull imaging in orthodontics. It is used for examination and diagnosis to formulate treatment plans as well as to monitor growth and progress of treatment. Facial forms can be quantitatively analyzed using linear, angular and ratio-based measurements [15]. However, it is unable to adequately describe the craniofacial morphology, such as size, shape, growth and changes in curvature [15,16,17]. Graphical shape analysis, as well as its illustration, is challenging because multiple superimpositions are impossible [18].

Geometric morphometric analysis (GMA) has gained prominence recently for its capacity to quantify the shapes of objects and offer a visual representation through statistical interpretation. This tool utilizes Cartesian landmark coordinates to investigate the variation in the shape of an object. It is centered on shape coordinates, effectively separating shape from overall size, position and orientation of landmark configurations, so the size of a structure does not introduce confounding factors in statistical analysis [19]. GMA enables the exploration of morphological variations, craniofacial growth, connections between morphology and various conditions and treatment outcomes. Within orthodontics, GMA can be applied in the shape and size analyses of the face. This analytical technique has been proven to be valuable for measuring facial and cranial structures, assessing facial proportions and understanding relationships between anatomical components [17]. GMA has been utilized in studies of growth patterns of hard tissue shapes across various facial types [17], variations in skeletal shape [19], soft tissue distinctions among different sagittal skeletal patterns [19], mandibular symphysis [20], arch forms [21] and palatal rugae [22].

Despite these applications, the use of GMA to evaluate hard and soft tissue shape changes, particularly in Class III malocclusion, remains limited. Although GMA is a cost-effective and powerful method for quantitative shape analysis, its adoption in orthodontic research has been constrained by several factors, including the need for specialized training to understand its theoretical concepts, the technical and technique-sensitive nature of landmark digitization and data interpretation and the higher cost and limited accessibility of three-dimensional imaging systems. Consequently, previous orthodontic studies have relied on simplified visualization methods such as lollipop and wireframe graphs, which may not fully or accurately represent complex shape differences. Therefore, this study aims to investigate the shape variations and changes in both hard and soft tissue in a Class III malocclusion before and after orthodontic treatment using GMA. This study may offer insights into the shape variations seen in Class III malocclusion and visually demonstrate the effects of orthodontic treatment on the maxilla, mandible and soft tissues, along with their interrelationships. The null hypothesis was that there are no significant hard or soft tissue shape variations in Class III malocclusion and there are no shape changes pre-treatment and post-treatment following orthodontic camouflage.

## 2. Materials and Methods

### 2.1. Samples

This retrospective study evaluated lateral cephalometric radiographs (LCRs) to examine the shapes and dimensions of hard and soft tissues in patients with Class III malocclusion before and near the completion of orthodontic treatment. Ethical approval was obtained from the Universiti Kebangsaan Malaysia (UKM) Research Ethics Committee (Reference: JEP-2024-534), dated 20 September 2024.

LCRs were collected from patients’ folders who had completed orthodontic treatment at the Department of Family Oral Health, UKM. Inclusion criteria were as follows: age 16–40 years; medically healthy; presence of all incisors; high-quality LCRs with clearly identifiable hard and soft tissue landmarks; and a skeletal Class III pattern (ANB < 2°) undergoing camouflage orthodontic treatment. Exclusion criteria were as follows: poor-quality LCRs; craniofacial or dental anomalies; cleft conditions; history of mandibular trauma or surgery; periodontitis; previous orthodontic treatment; or missing incisors.

There are currently no established guidelines or straightforward mathematical formulas for determining sample size in geometric morphometric analysis [20]. Therefore, a sample size calculation was made for linear measurement. To achieve 80% power at a 5% significance level to detect a mean paired difference of 5.47 with a standard deviation of 7.39, a minimum of 58 samples is required [23]. To account for potential exclusions due to poor-quality LCRs, 84 samples were ultimately included.

### 2.2. Lateral Cephalometric Radiograph Analysis

Pre-treatment (T1) and near-end treatment (T2) lateral cephalometric radiographs (LCR) were obtained from the Department of Radiology at Universiti Kebangsaan Malaysia. “Near-end treatment” was defined as radiographs obtained during the final phase of active orthodontic treatment, after active major tooth movement, mainly space closure and achievement of treatment objectives, but prior to appliance removal. A total of 168 LCRs were retrieved for both pre- and near-end treatment using Planmeca Romexis software (version 4.2, Planmeca ProMax 3D Classic; Planmeca Romexis, Helsinki, Finland). The exposure settings included 60–90 kV, 1–14 mA, an exposure time of 9 to 37 s and a 1.5 m distance between the source and the midsagittal plane. All lateral cephalometric radiographs were acquired using the same radiographic unit with a fixed source-to-mid-sagittal plane distance, resulting in a constant magnification factor across all images. All radiographs were obtained with patients in natural head position, teeth in maximum intercuspation and lips at rest. Standardized exposure parameters and head positioning protocols were used in accordance with institutional guidelines. Each patient’s radiographs were evaluated as paired observations for statistical analysis.

The LCRs were digitally traced using iOrtho (version 11.2.6, ©2025 Angelalign Technology, Shanghai, China). To ensure accuracy and consistency in tracing, all radiographs were adjusted so that the Frankfort horizontal plane served as the true horizontal reference for locating landmarks precisely.

**Figure 1:**
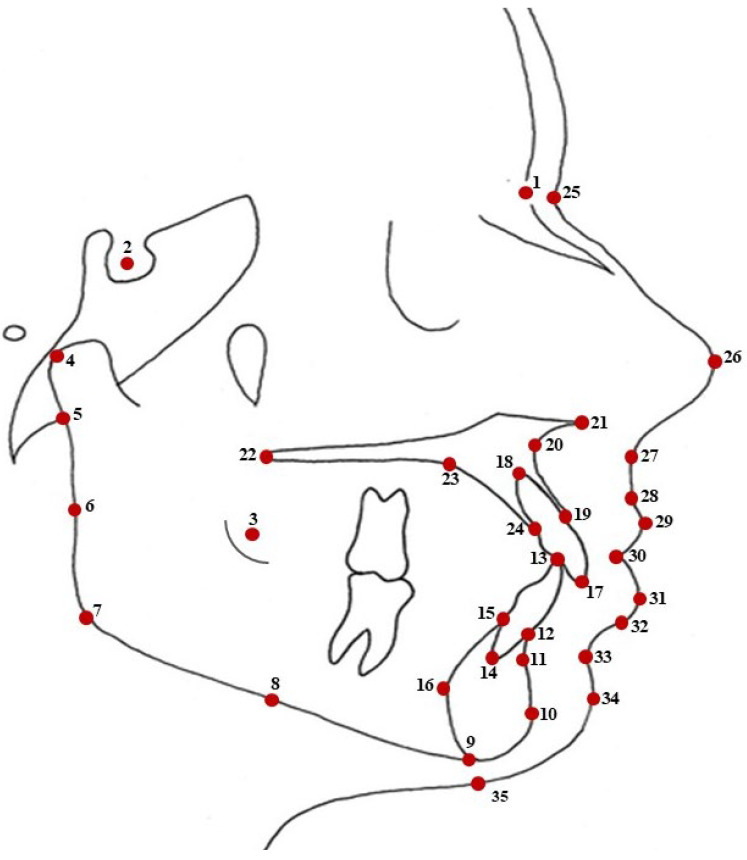
Landmarks for geometric morphometric analysis.

**Figure 2:**
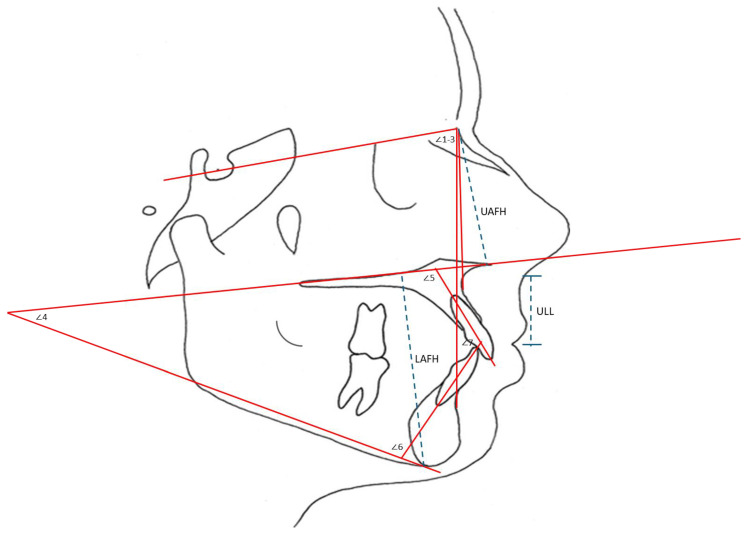
Angular (^o^) and linear (mm) measurements used in this study: SNA (∠1), SNB (∠2), ANB (∠3). Maxillary Mandibular Plane Angle (∠4), Upper Incisor Inclination (∠5), Lower Incisor Inclination (∠6), Interincisal Angle (∠7), Lower Anterior Face Height (LAFH), Upper Anterior Face Height (UAFH) and Upper Lip Length (ULL).

A total of 35 two-dimensional landmarks were identified, selected based on their relevance in traditional metrical and geometric morphometric analyses. These landmarks, commonly used in orthodontic assessments, provide significant biological reference points (Figure 1; Table 1). This is due to their anatomical stability, reproducibility and clinical relevance in evaluating Class III malocclusion. The landmark set was designed to comprehensively represent key skeletal anatomy of the cranial base, maxilla, mandible and dentoalveolar structures, as well as the corresponding soft tissue profiles, thereby allowing integrated assessment of hard–soft tissue relationships. Skeletal landmarks (Nasion, Sella, A-point, B-point, Gonion, Menton, Pogonion, ANS and PNS) capture sagittal and vertical discrepancies, while dentoalveolar landmarks (incisal and infradentale points) reflect orthodontic tooth movement. Soft tissue landmarks (Pronasale, Subnasale, Labrale superius and inferius, soft tissue Pogonion and Menton) were included to evaluate treatment-related facial profile changes. They were used to obtain linear and angular measurements (Figure 2) to evaluate hard and soft tissue changes after orthodontic treatment. During landmark digitization, all radiographs were anonymized and assessed in a randomized order, instead of in a chronological before-and-after sequence, to reduce examiner fatigue and minimize potential sequencing, recognition and recall bias inherent in retrospective longitudinal assessments.

LCR digital copies in JPG format were converted into TPS files using TPSUtil software (version 1.83, Rohlf) [24]. The landmark coordinate dataset (x, y) was generated with TPSDig2 software and subsequently imported into MorphoJ (version 2.0; Apache license, Klingenberg Lab, Manchester, UK) for shape analysis.

### 2.3. Shape and Statistical Analysis

For geometric morphometric analysis (GMA), all the shape and statistical analyses were conducted using MorphoJ software. Generalized Procrustes Analysis (GPA) was used to assess shape variations in the hard and soft tissues. Lateral cephalometric radiographs (LCRs) were superimposed, translated, scaled and rotated. This minimizes the total squared distances between corresponding landmarks and, as a result, establishes the general shape of the hard and soft tissues in the Class III malocclusion samples.

The primary objective of the study was to determine the presence of shape variations in hard and soft tissues at the pre-treatment stage (T1). This was performed using Principal Component Analysis (PCA). PCA reduced the dimensionality of the shape data and identified key variation patterns. The covariance matrix was then computed from the shape data, and its eigenvector, which is known as the principal component (PC), was extracted. The directions of the maximum shape variance in descending order were represented by each principal component.

A comparison of shape changes before (T1) and after (T2) orthodontic treatment was also carried out. Discriminant analysis (DA) was performed using a permutation test (1000 random permutations) to evaluate significant shape differences between repeated measurements of hard and soft tissues. This was crucial for minimizing digitization errors and subsequently introducing variance in the subsequent analysis. In addition, DA was also performed separately for the maxilla and mandible to enhance visualization. Procrustes distance was used to assess statistical differences at T2.

In addition to GMA, angular and linear measurements were taken to validate the changes detected in DA. Statistical Package for the Social Sciences (SPSS, version 25; SPSS, Chicago, IL, USA) was used to conduct dependent *t*-tests with a significance level of *p* < 0.05 and a 95% confidence level to compare the mean changes before and after orthodontic treatment. Descriptive statistics were calculated for each measurement at T1 and T2 and significant differences between them were tested using the dependent *t*-test if the data followed a normal distribution.

### 2.4. Reliability

Due to the retrospective study design, blinding of the examiner during landmark digitization and analysis was not feasible, as post-treatment radiographs were distinguishable by the presence of fixed orthodontic appliances. However, standardized landmark definitions and repeated digitization were employed to minimize potential observer bias. Therefore, intra-examiner and inter-examiner reliability tests were carried out on 10 randomly selected cases 1 month apart. The tests were performed using the Interclass correlation coefficient test (ICC) on tracing measurements. The correlation coefficients for intra-examiner were above 0.9996 and 0.998 for inter-examiner reliability. Both results were more than 0.85, indicating excellent agreement between measurements. For GMA, measurement error from landmarking was checked with Procrustes ANOVA using MorphoJ software. The F-ratio for this effect showed that individual ANOVA variation was 1587 times larger than the measurement error due to digitizing for centroid size and 13 times for shape. This indicates that the individual variation exceeded the measurement error due to digitizing (F > 1). In addition, the discriminant analysis indicated no significant difference in shape distance between repeated measurements of specimens (*p* > 0.05).

## 3. Results

There were 84 samples comprising 52 females (62%) and 32 males (38%) with a mean age of 24.7 ± 5.2. The majority of the patients were Malay (64%), followed by Chinese (33%) and Indian (3%). Furthermore, 9 (11%) were adolescents, 32 (38%) were emerging adults and 43 (51%) were adults. The majority of the samples, 43 (51%), had an average MMPA, whereas 24 (29%) and 17 (20%) had low angle and high angle, respectively.

**Figure 3:**
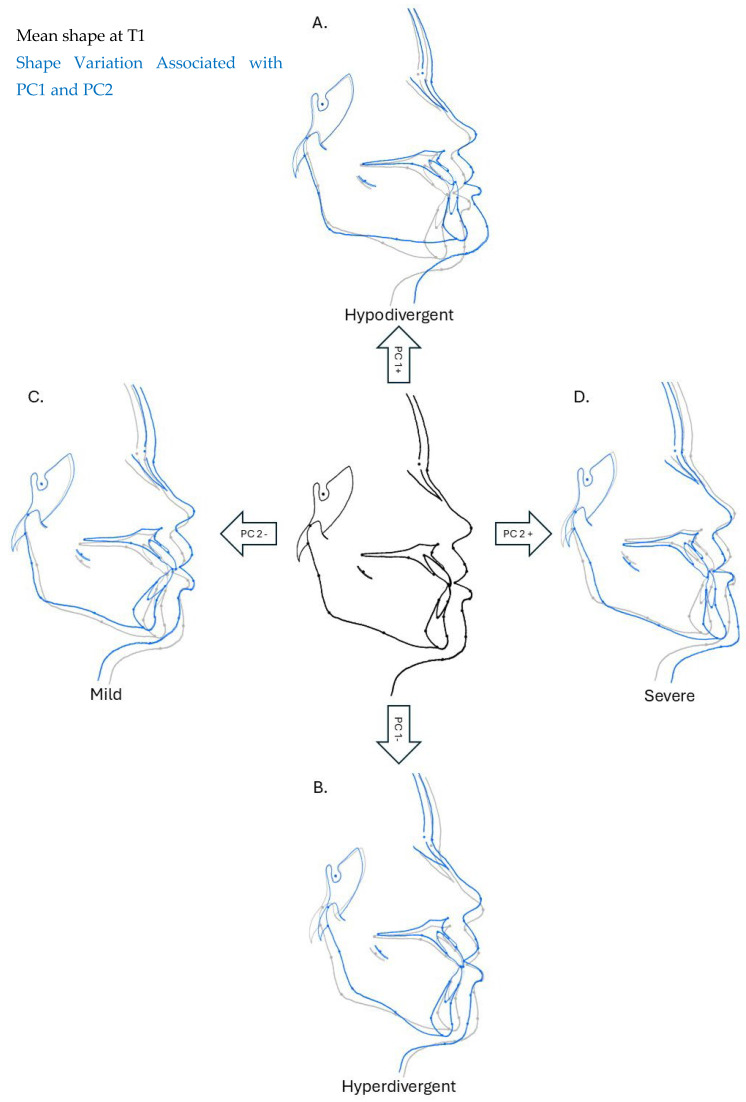
Hard and soft tissue shape variations in vertical and anteroposterior dimensions. Black and gray outline: Overall mean shape at pre-treatment. Blue outline PC1 (**A**,**B**): Vertical shape variation. Blue outline PC2 (**C**,**D**): Anteroposterior shape variation.

Principal Component Analysis (PCA) revealed that facial skeletal morphology in Class III patients varies mainly in the vertical (PC1) and anteroposterior (PC2) dimensions. In this study, PCA yielded 66 dimensions of hard and soft tissue shape variations, represented by PC1 to PC66. PC1 and PC2 represented the two highest variations within the whole data. PC1 (23.35%) showed the highest variations and described them in the vertical dimension; the facial profile varied from hypodivergent to hyperdivergent. The maxilla mandibular plane angles and gonion angle varied from acute to obtuse. The upper lip lengths varied from short to long. PC2 (13.51%), on the other hand, described variations in the anteroposterior dimension, where the severity of the Class III skeletal pattern varied from mild to severe. The facial profile varied from straight to concave, while the labio-mental fold varied from increased to reduced. The lower incisors differed from retroclined to proclined, while the overjet was either positive or negative (Figure 3).

**Figure 4:**
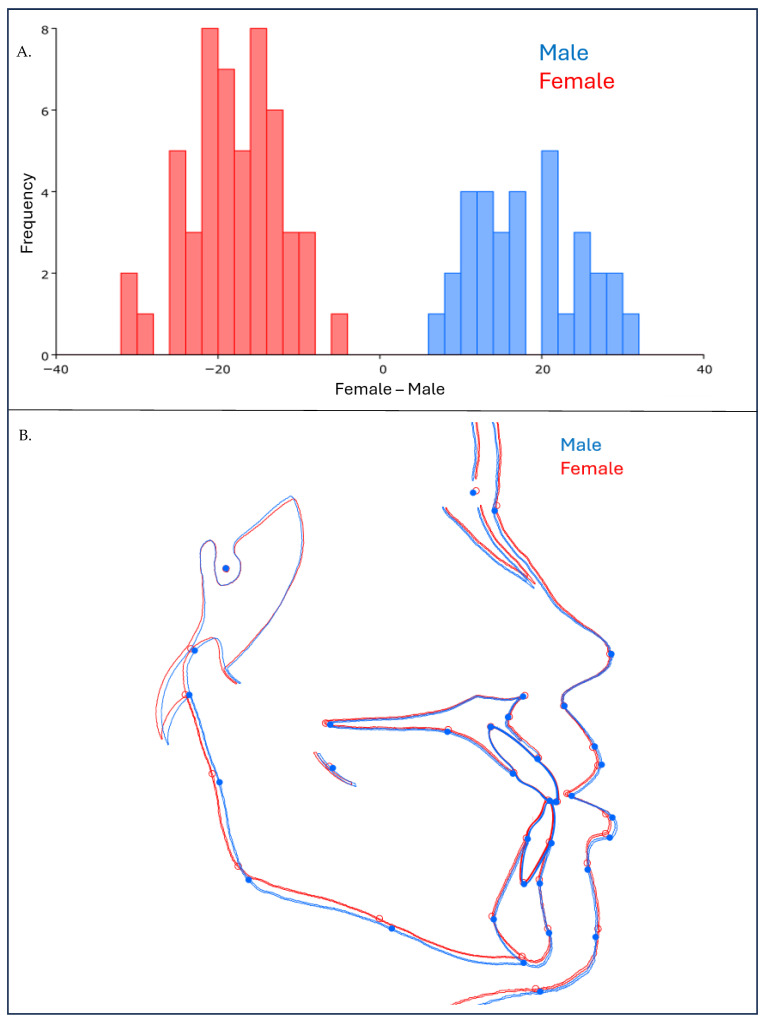
Discriminant analysis (DA) of hard and soft tissue comparing males and females before orthodontic treatment. (**A**) Analysis graph of male (blue) and female (red) (**B**) warped outline drawing of male (blue) and female (red). Overlapping regions indicate minimal morphological differences, while deviations highlight significant morphological differences.

**Figure 5:**
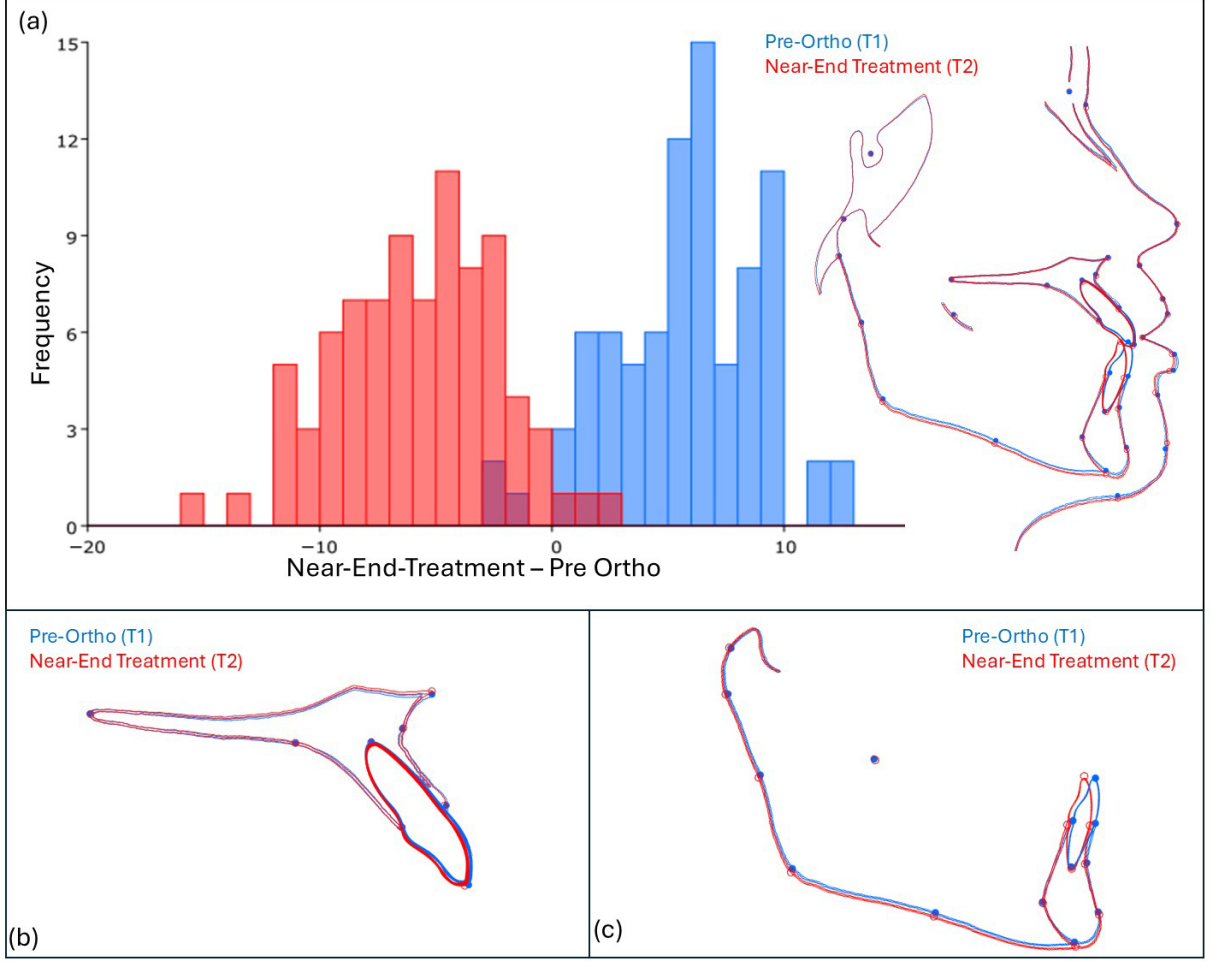
Discriminant analysis (DA) of hard and soft tissue before (T1—blue) and near-end orthodontic treatment (T2—red). Overlapping regions indicate minimal morphological change, while deviations highlight significant morphological shifts near-end treatment. (**a**) Overall analysis graph and warped outline drawing comparing pre-treatment (T1—blue) and near-end treatment (T2—red) shapes. (**b**) DA focused on the maxilla, showing stability or changes between T1 (blue) and T2 (red). (**c**) DA of the mandible, demonstrating significant shape changes post-treatment (T2—red) compared to pre-treatment (T1—blue).

Table 2 shows that, among the tested explanatory factors, gender accounted for the largest proportion of explained shape variance (%SS = 30.91%) with a strong effect and significant *p*-value (F = 56.99, *p* < 0.0001), followed by race (%SS = 9.24%, F = 8.52, *p* = 0.0008) and age group (%SS = 6.51%, F = 6, *p* = 0.0051). Meanwhile, individual variation accounted for the greatest overall proportion of shape variance (%SS = 51.90%). In contrast, MMPA explained only a small proportion of shape variance (%SS = 1.44%) and did not reach statistical significance (*p* = 0.3536). Figure 4 shows the analysis graph and warped outline drawing of the hard and soft tissues, comparing males and females before orthodontic treatment. The Procrustes distance (PD) between the mean shapes of males and females was 0.026 and the permutation test showed significant shape variations between males and females (*p* < 0.05) (Figure 4). Overall, the males had larger and longer faces. The ramus, body of the mandible and alveolar height in males were significantly longer than in females (*p* < 0.05) (Figure 5). Males’ LAFH was significantly longer (66.41 ± 4.99 mm) than in females (60.42 ± 4.56 mm, *p* < 0.05). The TAFH in males was significantly longer (119.04 ± 6.26 mm) compared to females (109.55 ± 5.62 mm, *p* < 0.05). Males have longer upper lip length (22.92 ± 1.92 mm) compared to females (20.31 ± 2.20 mm, *p* < 0.05) (Table 3).

Figure 5 shows Discriminant analysis (DA) of hard and soft tissue before (T1) and near-end orthodontic treatment (T2). The Procrustes distance between the overall mean shapes of T1 and T2 was 0.019, and permutation test showed significant shape differences (*p* < 0.05). In the mandible, the red outline (T2) deviates noticeably from the blue outline (T1), indicating significant morphological changes. This is supported by statistical analysis, where the Procrustes distance between T1 and T2 was 0.018 (*p* < 0.05). In the maxilla, blue and red outlines closely overlap, suggesting minimal change at near-end treatment.

There was a significant reduction in the SNB by 0.41° and ANB by 0.46° (*p* < 0.05) and in the retroclination of lower incisors by 4.97° (*p* < 0.05), and there was an increase in inter-incisal angle by 5.9° (*p* < 0.05). The lower anterior facial height (LAFH) increased by 1.78 mm (*p* < 0.05), while the total anterior facial height (TAFH) also increased by 2.63 mm (*p* < 0.05). There was an increased upper lip length after treatment by 0.40 mm (*p* < 0.05) (Table 4).

## 4. Discussion

### 4.1. General Shape Variation in Hard and Soft Tissue of Class III Malocclusion

PCA revealed that the most significant variation was in the vertical dimension, followed by anteroposterior dimensions, which explains the heterogenicity in Class III malocclusion [3,25]. It also aligns with a study stating that there is a range from mild to severe of skeletal patterns within this class of malocclusion [25]. Vertical assessment must be emphasized as it influences the treatment plan and mechanics. Clinically, the vertical growth pattern and severity of skeletal discrepancy play an important role in determining the complexity and planning of orthodontic treatment. Anteroposteriorly recognizing the severity of the skeletal Class III is important as it directly influences the selection of appropriate orthodontic or surgical interventions.

Mild–moderate skeletal discrepancies may be addressed with orthodontic camouflage or growth modification techniques, whereas moderate to severe cases often require orthognathic surgery to achieve optimal functional and esthetic results [26]. A hyperdivergent profile is associated with increased anchorage demands, and the loss of anchorage during treatment can adversely affect outcomes. In addition, Class III is often associated with an open bite and this further complicates treatment as it often focuses on reducing vertical height through posterior teeth intrusion to encourage counterclockwise mandibular rotation, which can be very challenging and unpredictable [11]. Nonetheless, this approach not only improves facial esthetics but also enhances occlusal function. Therefore, meticulous planning, especially concerning extractions, anchorage requirements and mechanics, is essential [27].

### 4.2. Effect of Gender on Size and Shape of Hard and Soft Tissue of Class III Malocclusion

Males demonstrated significantly larger craniofacial dimensions compared to females (*p* < 0.05), including greater TAFH, LAFH, mandibular ramus, body, alveolar height and upper lip length. These differences align with established anthropological findings [28,29,30,31].

Gender-based morphological differences must be considered during treatment planning, particularly regarding anchorage requirements and biomechanical responses, especially in the vertical dimension. Males’ larger skeletal dimensions may require modified force systems and anchorage strategies. Additionally, esthetic treatment goals may differ between genders, necessitating individualized soft tissue targets that account for these inherent dimorphic characteristics [8,32].

### 4.3. Effect of Treatment on Pre- and Near-End Treatment of Hard and Soft Tissue Size and Shape

Significant hard and soft tissue changes were observed post-treatment. SNB reduced while ANB improved slightly (*p* < 0.05), indicating an elimination of mandibular displacement rather than a true skeletal change. Hence, patients usually remain in a Class III skeletal pattern. Crossbite correction and mandibular displacement elimination caused the mandible to position slightly posteriorly, resulting in an increase in TAFH and LAFH (*p* < 0.05) [33]. The maxilla remained stable as expected in camouflage treatment of non-growing patients (*p* > 0.05) [33]. Lower incisors were retroclined by 4.97° (*p* < 0.05), while upper incisors maintained their proclination (*p* > 0.05) to achieve a positive overjet and average overbite, thus causing an increase in interincisal angle (*p* < 0.05). This is consistent with orthodontic camouflage principles and usually associated with the extraction of the lower first premolars to achieve Class I malocclusion [34,35,36]. These findings reinforce the fact that camouflage therapy primarily induces dentoalveolar changes rather than substantial skeletal correction [4].

In general, the greater the degree of proclination or retroclination of a tooth, the higher the risk of relapse. Mills (1968) reported that retroclination of 7° or more may result in up to 50% relapse [37]. In the present study, the observed relapse potential was less than 50%, indicating favorable post-treatment stability. Nevertheless, compliance with retainer wear remains essential, and bonded retainers are warranted in cases involving severe rotations or spacing [38]. In addition, forces from the tongue may contribute to the proclination of the lower incisors, thereby increasing the risk of relapse [39]. Esthetically, lower lip retrusion may follow incisor movement at a 1.3:1 ratio [36], improving the facial profile. However, excessive lower incisor retroclination can create an aged appearance through loss of lower lip support and increased mentolabial fold depth. Clinicians must balance skeletal correction with maintenance of adequate lip support to optimize both dental and facial esthetics [40,41].

### 4.4. Geometric Morphometric Analysis (GMA)

The present study demonstrates that GMA serves as a robust analytical tool with significant potential in clinical applications such as orthognathic surgery planning as well as facial asymmetry and airway assessment. Moreover, GMA can be incorporated with digital and three-dimensional imaging. This will significantly benefit the management of complex cases as it provides detailed analysis and insights into such conditions.

In this context, GMA has been demonstrated to be more effective than conventional two-dimensional techniques in capturing detailed variations in hard and soft tissue morphology. This study has shown that GMA enables a more comprehensive evaluation of soft tissue structures by maximizing the information obtained from lateral cephalometric radiographs routinely used in orthodontic practice. Although certain soft tissue regions reflect underlying skeletal changes, it is well recognized that not all soft tissue components correspond directly to the skeletal profile [42].

Beyond analytical advantages, GMA also facilitates patient communication by providing visual illustrations of skeletal and facial shape variation, enabling patients, particularly those with severe Class III malocclusion, to better understand their underlying facial morphology. Furthermore, the success of orthognathic surgery can be evaluated by analyzing skeletal and soft-tissue shape changes relative to pre-treatment morphology.

From a technical perspective, the majority of cephalometric software only enables up to three LCR superimpositions. GMA allows multiple LCR superimpositions of landmark configurations, which allow assessment of treatment outcomes by creating an average shape. This is a good tool for orthodontists or oral maxillofacial surgeons to assess immediate post-orthodontic treatment changes and orthognathic surgery within a population.

Finally, GMA facilitates the evaluation of long-term stability and relapse following orthodontic treatment and orthognathic surgery. By offering an objective framework for outcome evaluation, GMA supports continuous refinement of both surgical and orthodontic techniques. Such an approach is increasingly important in meeting the expectations of esthetically driven and highly demanding patients [43].

### 4.5. Limitations

One of the limitations of this study is the retrospective nature of the study and the lack of a surgical control group limits control over data acquisition, which may introduce selection bias. Future studies using a randomized clinical trial design that includes an orthognathic surgery group may better elucidate dentoalveolar and skeletal changes. Furthermore, the two-dimensional nature of the lateral cephalometric analysis precluded assessment of transverse and volumetric craniofacial changes, potentially overlooking clinically relevant asymmetries and lateral discrepancies. Three-dimensional imaging, such as cone-beam computed tomography (CBCT), would provide more comprehensive insights into three-dimensional craniofacial variation and treatment modalities. The sample exhibited limited ethnic diversity and an unbalanced ethnic distribution, which may limit the generalizability of the findings to other populations. The results should therefore be interpreted primarily within the context of Asian populations. Therefore, future studies should incorporate three-dimensional morphometric approaches, balanced multi-ethnic samples, surgical comparison groups and longitudinal follow-up to evaluate long-term treatment stability.

## 5. Conclusions

The null hypothesis was rejected, as orthodontic camouflage treatment resulted in statistically and morphologically significant hard and soft tissue shape changes. This study demonstrates that geometric morphometric analysis (GMA) is a cheap and clinically valuable supportive tool for evaluating hard and soft tissue shape variation and treatment-related changes in Class III malocclusion. The findings indicate that orthodontic camouflage produces acceptable skeletal and dental compensation. While soft tissue adaptations remain modest and relatively stable, improvements in facial profile were observed despite persistence of underlying skeletal discrepancy. Clinically, GMA enables objective visualization and quantification of craniofacial changes. A combination of GMA and LCRs analyses facilitates more informed treatment planning and decision-making, particularly in borderline cases. Therefore, incorporating both hard and soft tissue analysis, such as GMA, will enable clinicians to diagnose and formulate a customized orthodontic and surgical treatment plan, monitor its changes and reflect on treatment modalities. Furthermore, clinician–patient communication can be improved, enabling a more holistic approach to care while avoiding the overestimation of treatment outcomes or the underestimation of procedural complexity.

## Figures and Tables

**Table 1:** Landmarks used for geometric morphometric analysis, data from ([15]).

No.	Name	Description
1	Nasion	The most anterior point on the frontonasal suture in the midsagittal plane
2	Sella	The midpoint of the pituitary fossa or sella turcica
3	Ab	Anterior border of the ramus
4	Condylion	The most lateral point on the surface of the condyle of the mandible
5	Articulare	The point of intersection of the external dorsal contour of the mandibular condyle and the temporal bone
6	Pb	Posterior border of the mandible between Ar and Go
7	Gonion	The most posterior and inferior point on the bony chin
8	Mandible point	The instrumentally determined mid-point of the mandible between the gonion and the menton
9	Menton	The most inferior point of the mandibular symphysis
10	Pogonion	The most prominent point of the mandibular symphysis
11	B-Point	Deepest point on the curved profile of the mandible between the chin and alveolar crest
12	Mn Anterior Infradentale	Highest anterior point of the mandibular alveolar bone
13	LiT	Incisor tip of mandibular incisors
14	LiA	Root apex of mandibular incisors
15	Mn Posterior Infradentale	Highest posterior point of the mandibular alveolar bone
16	Pg’	The most prominent point of posterior symphysis
17	UiT	Incisal edge of maxillary incisors
18	UiA	Root apex of maxillary incisors
19	Prosthion	The point of the upper alveolar process that projects most anteriorly
20	A-Point	The deepest point on the contour of the maxillary alveolar process
21	ANS	Anterior nasal spine
22	PNS	Posterior nasal spine
23	SAHP	The most superior and anterior points of the hard palate
24	Mx Posterior Infradentale	The intersection of the alveolar bone of the maxilla with the palatal surface of the maxillary incisor
25	Soft Tissue Nasion	The point of the greatest concavity in the midline between the forehead and the nose
26	Pronasale	The most prominent or anterior point of the nose
27	Subnasale	The junction between the lower border of the nose and the beginning of the upper lip
28	Upper Vermillion Border	The junction between the oral mucosa and the adjacent facial skin of the upper lip
29	Labrale Superius	The most anterior point on the margin of the upper membranous lip
30	Lips Contact Point	Contact or the shortest distance between the upper and lower lips
31	Labrale Inferius	The most anterior point on the margin of the lower membranous lip
32	Lower Vermillion Border	The junction between the lower lip vermillion and the facial skin
33	Sulcus Inferius	The point of greatest concavity in the midline of the lower lip between the labrale inferius and the soft tissue pogonion
34	Soft Tissue Pogonion	The most prominent point of the soft tissue chin
35	Soft Tissue Menton	A point on the soft tissue chin from vertical extension from the menton

**Table 2:** Effects of gender, age, race and MMPA on the size of hard and soft tissues.

Effect	%SS	SS	MS	df	F	*p*
Race	9.24	12.76	6.3809	2	8.52	0.0008 *
Gender	30.91	42.67	42.67165	1	56.99	<0.0001 *
Age Group	6.51	8.98	4.49154	2	6	0.0051 *
MMPA	1.44	1.99	0.99384	2	1.05	0.3536
Individual	51.9	71.66	0.94285	76		

Procrustes ANOVA. * *p* < 0.05 was considered statistically significant

**Table 3:** Lateral cephalometric measurements in males and females.

Measurements	Male	Female	Mean Difference	Cohen’s d	95% Confidence Interval	*p*-Value
	Mean ± SD (°)	Mean ± SD (°)			(Lower, Upper)	
SNA	81.38 ± 3.18	80.66 ± 3.09	0.73	0.23	(−0.21, 0.67)	0.30
SNB	82.20 ± 4.12	81.44 ± 3.37	0.76	0.20	(−0.24, 0.64)	0.36
ANB	−0.82 ± 2.12	−0.79 ± 1.74	−0.03	−0.016	(−0.46, 0.43)	0.94
MMPA	25.53 ± 6.55	25.69 ± 5.28	−0.16	−0.028	(−0.47, 0.41)	0.90
UiMX	120.72 ± 7.15	120.38 ± 7.17	0.33	0.05	(−0.39, 0.49)	0.84
LiMn	92.74 ± 8.17	92.34 ± 8.87	0.40	0.05	(−0.39, 0.48)	0.84
IIA	123.16 ± 14.14	123.68 ± 11.25	−0.52	−0.042	(−0.48, 0.40)	0.85
FH Ratio	55.75 ± 2.26	55.20 ± 1.85	0.56	0.28	(−0.17, 0.72)	0.22
LAFH	66.41 ± 4.99	60.42 ± 4.56	5.99	1.25	(0.76, 1.72)	0.00 *
TAFH	119.04 ± 6.26	109.55 ± 5.62	9.50	1.62	(1.11, 2.12)	0.00 *
ULipLength	22.92 ± 1.92	20.31 ± 2.20	2.61	1.25	(0.76, 1.72)	0.00 *

Independent *t*-tests. * *p* < 0.05 was considered statistically significant

**Table 4:** Lateral cephalometric pre-treatment and near-end treatment.

Measurement	Pre-Treatment (T1)	Near-End-Treatment (T2)	Mean Differences	Cohen’s d	95% Confidence Interval	*p*-Value
	Mean ± SD (°)	Mean ± SD (°)			(Lower, Upper)	
SNA	80.93 ± 3.12	80.97 ± 3.12	−0.04	−0.035	(−0.25, 0.18)	0.75
SNB	81.73 ± 3.67	81.32 ± 3.57	0.41	0.37	(0.15, 0.59)	0.00 *
ANB	−0.80 ± 1.88	−0.33 ± 1.82	−0.46	−0.45	(−0.69, −0.22)	0.00 *
MMPA	25.63 ± 5.76	26.73 ± 10.83	−1.10	−0.13	(−0.34, 0.088)	0.25
UiMx	120.51 ± 7.12	118.48 ± 11.66	2.04	0.19	(−0.031, 0.40)	0.09
LiMn	92.50 ± 8.56	87.53 ± 6.96	4.97	0.63	(0.40, 0.86)	0.00 *
IIA	123.48 ± 12.35	129.38 ± 9.27	−5.90	−0.45	(−0.69, −0.22)	0.00 *
FH Ratio	55.41 ± 2.02	55.34 ± 2.81	0.07	0.03	(−0.18, 0.25)	0.77
LAFH	62.70 ± 5.53	64.48 ± 5.76	−1.78	−0.57	(−0.80, −0.34)	0.00 *
TAFH	113.16 ± 7.45	115.79 ± 7.88	−2.63	−0.53	(−0.76, −0.30)	0.00 *
ULipLength	21.30 ± 2.44	21.70 ± 2.46	−0.40	−0.22	(−0.44, −0.005)	0.04 *

Dependent *t*-test. * *p* < 0.05 was considered statistically significant

## Data Availability

The raw data supporting the conclusions of this article will be made available by the authors on request.
